# In situ measurement of the specific surface area of reduced graphene oxide using time-resolved laser induced incandescence

**DOI:** 10.1007/s00340-026-08633-0

**Published:** 2026-04-11

**Authors:** Horace I. Looi, Sarah Jankhani, Michael Pope, Kyle J. Daun

**Affiliations:** 1 Department of Mechanical and Mechatronics Engineering, University of Waterloo, Waterloo, ON Canada; 2 Department of Chemical Engineering, University of Waterloo, Waterloo, ON Canada

## Abstract

Reduced graphene oxide (rGO) produced by the rapid thermal expansion of graphite oxide is a promising alternative to graphene for applications requiring large volumes and some chemical functionality on the graphene, since this approach offers potentially high yields and throughput. However, the surface area and single layer dispersibility of the resulting rGO powder depends heavily on precursor moisture content, chemistry and morphology, as well as and process parameters that include residence time and quenching rates. While large-scale commercial deployments have been demonstrated, there are currently no real-time diagnostics capable of assessing inline material quality. In this study, we propose time-resolved laser-induced incandescence (TiRe-LII) as a means to derive the real-time relative specific surface area (*S*_sp_) of rGO particles which is expected to be a strong indicator of single sheet dispersibility. The TiRe-LII derived *S*_sp_ were found to be consistent in both magnitude and trend to those found through batch gas adsorption measurements analyzed by fitting the Brunauer-Emmett-Teller (BET) isotherm.

**Supplementary Information:**

The online version contains supplementary material available at 10.1007/s00340-026-08633-0.

## Introduction

Graphene-based materials are increasingly sought for use in electrical, biomedical, and mechanical applications. In energy-storage devices like batteries and supercapacitors, pristine single layer graphene (PG) is particularly valued due to its high electron and hole mobility, which can lead to high conductivity when sufficiently doped or gated, as well as its mechanical and electrochemical stability [1]. The most prominent methods of synthesizing PG, such as chemical vapor deposition (CVD) and epitaxial growth, are inherently batch processes that are difficult to scale up to produce industrially relevant quantities [2]. While continuous roll-to-roll CVD reactors have been built that can deposit large films continuously, they cannot produce the bulk quantifies of materials necessary for applications such as polymer composites or electrodes for batteries or supercapacitors. This limitation has motivated research into PG-like alternatives that are more amenable to high yield and high-volume production.

Of these candidates, reduced graphene oxide (rGO) is particularly promising due to its comparable electrical properties to PG, amenability to large scale production, and tunability in terms of the residual degree of oxidation, defects, and dopants [3–5]. This material is produced by first synthesizing an intermediate material, graphene oxide (GO), usually through oxidation and exfoliation of graphite [6]. Graphene oxide can then be chemically, electrochemically, or thermally reduced to form rGO. Solution-based reduction of colloidally dispersed GO yields precipitates of randomly aggregated thin, crumpled sheets of rGO with conductivity comparable to PG [7]. Synthesis routes involving colloids are inherently batch processes, however, which imposes a limit on the production rate. Likewise, electrochemical reduction of GO films can produce rGO coatings having high conductivity, but, again, through a batch process [8].

Rapid thermal expansion of graphite oxide, in contrast, is well-suited to continuous, large-scale production [9]. In principle, an aerosolized graphite oxide powder can be flowed through the hot zone of a furnace, resulting in the continuous production of a high surface area rGO powder aggregate. The temperature and residence time dictate the resulting C/O ratio of the material which offers tunability of the resulting rGO properties. Schniepp et al. [10] showed that thermal exfoliation and reduction of dry, agglomerated graphene oxide (GO) powders into exfoliated rGO sheets can be achieved by the rapid thermal expansion and simultaneous thermal reduction of GO powder in a quartz tube by rapidly inserting the tube into a furnace preheated to 1050 °C. The rapid exothermic decomposition of the functional groups on the GO into gaseous products (e.g., CO, CO_2_ and H_2_O) at a timescale faster than diffusion of the gases out of the inter-gallery spaces causes a pressure build-up and rapid deflagration of the material—effectively blowing the sheets apart [11]. Further academic studies [12, 13] showed that aerosolized GO powder can be transported into a heated tube furnace to rapidly produce exfoliated rGO particles. Whether batch or continuous production, after the powder is dispersed within a solvent, the resulting sheets of single and few-layer rGO exhibit a wrinkled or crumpled morphology having higher specific surface areas compared to flat sheets, a desirable attribute in many applications such as energy storage. Additionally, the tunability of rGO characteristics during thermal reduction have been shown to depend primarily on the reduction temperature [14], which may be leveraged to adjust the rGO properties, and hence its functionality. Given its tunability and fast processing, thermal reduction holds significant promise for large scale synthesis. However, to help foster industrial adoption of these materials, consistency and reproducibility in production is of paramount importance. This requires methods for process monitoring and control—ideally those amenable to continuous, inline monitoring. To date, no studies have investigated real-time diagnostics to monitor the functional properties of rGO during production.

One important metric for the resulting powder is the specific surface area (*S*_sp_) which would impact the obtainable single layer yield once the exfoliated product is dispersed within a solvent or composite matrix. This parameter provides information on particle size, packing density, and surface characteristics. Current methods of measuring *S*_sp_ require batch sampling, like gas absorption via Brunauer-Emmett-Teller (BET) theory, which is time consuming, expensive, and incompatible with real-time, online analysis.

In this context, time-resolved laser-induced incandescence (TiRe-LII) shows considerable promise. This diagnostic is most often associated with measurements of soot primary particle size and volume fraction in combustion-related applications [15]. It uses a pulsed laser to heat nanoparticles within a probe volume to incandescent temperatures, which is measured, usually at multiple wavelengths, as the particles return to their resting temperature. The particle volume fraction may be inferred by comparing the peak intensity signal with the pyrometrically-inferred temperature, while the particle size is connected to the signal decay rate by a heat transfer model of the cooling process.

While TiRe-LII is mainly considered a combustion diagnostic for measuring soot-laden aerosols, it is increasingly applied to characterize engineered nanoparticles as they are synthetized, without requiring physical access to the aerosol. Musikhin et al. [16] used TiRe-LII to measure aerosolized few-layer graphene (FLG) grown by plasma-induced decomposition of ethanol and found that FLG has higher absorption characteristics than soot and its signal intensity is linearly dependent on particle concentration. In a later study, López-Cámara et al. [17] measured the *S*_sp_ of FLG using TiRe-LII in real-time directly after synthesis in a plasma reactor. They found TiRe-inferred *S*_sp_ of the gas-phase particles to match those derived from BET analysis of the powder.

This study examines the viability of utilizing TiRe-LII to measure the *S*_sp_ of rGO, as part of a planned online processing system. The TiRe-LII system is placed at the exit of a tube furnace used to transform an aerosol of GO particles into rGO particles. The particle *S*_sp_ values are inferred from LII signal decay and compared to those found through BET analysis. Additionally, rGO particles with different degrees of reduction are synthesized by varying the reduction temperature and their *S*_sp_ values are compared to literature results to help elucidate the change in morphology. The *S*_sp_ determined from TiRe-LII is consistent in both magnitude and trend to those from BET analysis, although uncertainty in the density and specific heat of the rGO particles limits the quantitative comparison.

## Experimental methods

### GO powder synthesis and rGO thermal reduction

GO was synthesized by modified Hummer’s method [18] using sulfuric acid (H_2_SO_4_; Sigma-Aldrich) and phosphoric acid (H_3_PO_4_; ≥85 wt% in water, Sigma-Aldrich) with volume ratio of 9:1 for a total volume of 800 mL to oxidize 6 g of graphite flakes (Alfa Aesar, − 10 mesh, 99% metals basis). Thirty-six grams of potassium permanganate (ACS, LabChem) was introduced portion by portion into the acid mixture under stirring, followed by the graphite. The slurry was heated to 51 °C for 17 h. Once the reaction was completed and the slurry had cooled down, 800 g of ice was added gradually to dilute the slurry, followed by ~ 20 mL of hydrogen peroxide (H_2_O_2_; 30 wt% in water, Sigma-Aldrich) under stirring until the color of the slurry changed from dark purple to yellow. The slurry was then centrifuged, supernatant discarded, and the pellet resuspended in 10% hydrochloric acid (HCl; Sigma-Aldrich). This procedure was repeated twice with HCl followed by four washes with ethanol to remove residual HCl.

To prepare the GO powder, the synthesized graphene was dispersed in ethanol at a concentration of 12 g GO per liter of ethanol. The suspension was stirred overnight and then tip-sonicated (Sonics Vibra-Cell) for 10 min at 60% power. Afterwards, the suspension was spray-dried using a Büchi B-290 mini spray dryer at an inlet temperature of 180 °C, 30% pump power, 100% aspirator, and a nitrogen gas flow of 600 L h⁻¹. A Büchi inert loop B-295 system was employed to maintain oxygen-free conditions, prevent explosion hazards, and recapture ethanol in the condensing system.

Scanning electron microscopy (SEM; Hitachi SU8600) and transmission electron microscopy (TEM; Hitachi HT7830 operating at 100 keV) were conducted to analyze the structure of the GO powder. GO was deposited on a silicon wafer using the Langmuir–Blodgett technique, and atomic force microscopy (AFM; Veeco, USA) was performed using NP-STT10 tips (Bruker, USA) was used to characterize the thickness of the GO sheets. X-ray diffraction (XRD; Rigaku Miniflex II) was performed over a range of 3° to 50° to investigate the crystal structure of the material.

**Fig. 1:**
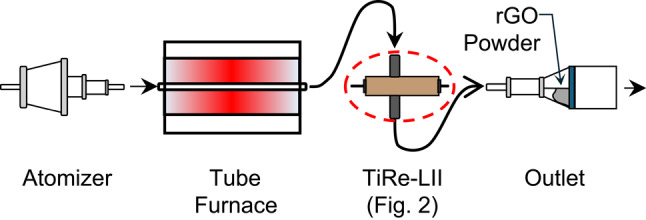
Integration of the aerosol generator, tube furnace, LII system, and outlet/filter

Figure 1 shows a schematic of the experimental apparatus. The GO powder was aerosolized using a custom dry powder nebulizer consisting of a semipermeable filter and a 2.8 W computer fan. The powder was placed onto a semi-permeable filter, where incoming argon gas at a rate of 8.50 standard litres per minute entrained the powder, creating an aerosol with assistance from the fan. The aerosol was then directed through a tube furnace (Carbolite STF 16/180), consisting of silicon carbide elements arranged around a ceramic tube [19]. The furnace was preheated to a set temperature and soaked for 30 min prior to flowing any aerosol through the furnace. The aerosol leaving the tube furnace was directed to a second semi-permeable filter where the rGO powder could be collected and the argon gas could be exhausted. During this study, five furnace set points were chosen as 500 °C, 700 °C, 900 °C, 1100 °C, and 1300 °C to monitor the change in *S*_sp_ versus reduction temperature, using the same GO powder, motive gas, and flow speed in all cases.

### Time-resolved laser-induced incandescence apparatus

**Fig. 2:**
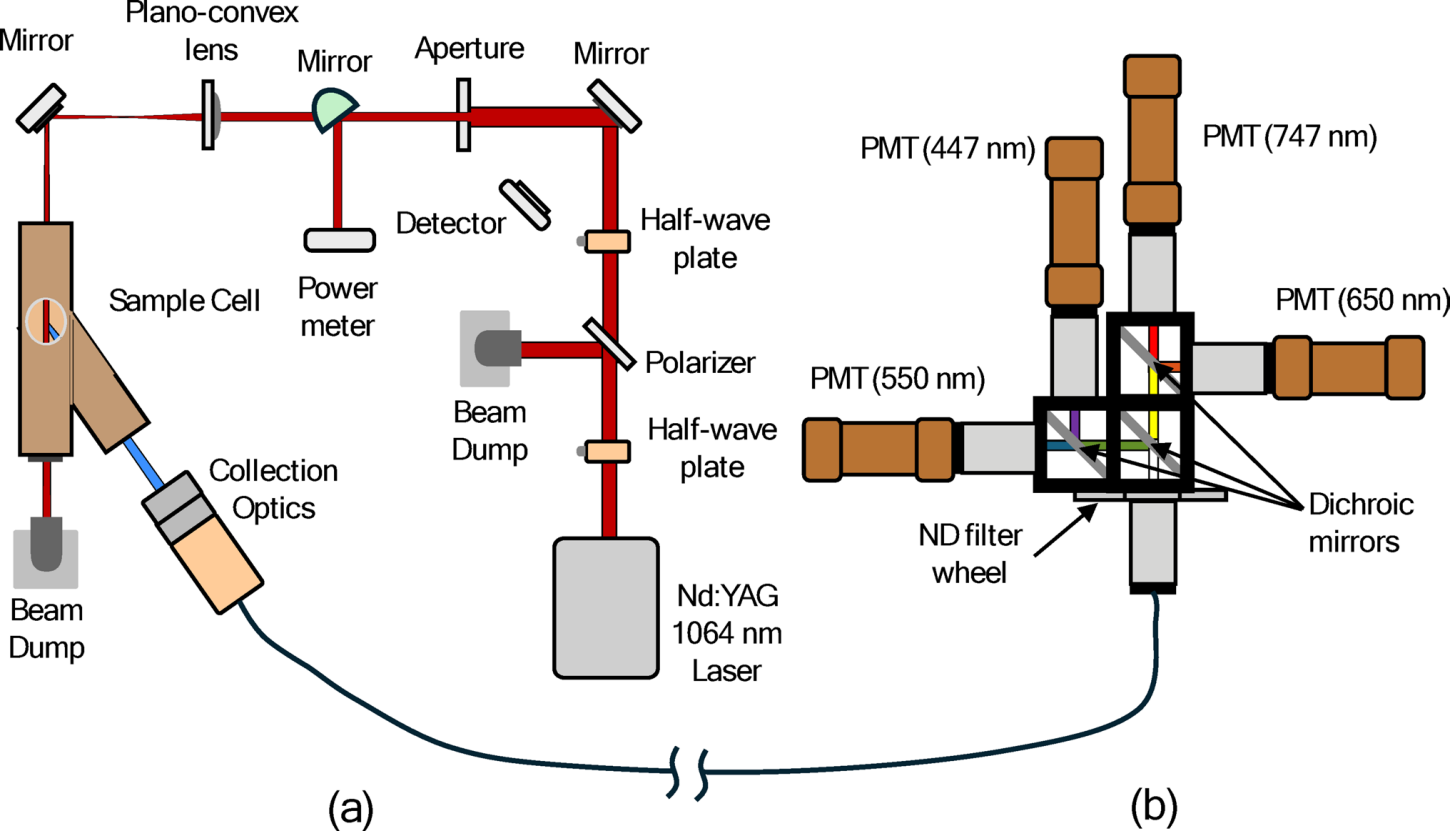
Schematic of TiRe-LII system:** a** excitation module;** b** detection module

Figure 2 shows a schematic of the TiRe-LII system used to characterize the rGO, which is composed of excitation and detection modules. Figure 2a shows the excitation module, consisting of a pulsed neodymium-doped yttrium aluminum garnet (Nd: YAG) laser centered at 1064 nm, beam conditioning optics, a sample cell where the aerosol is introduced, and collection optics. The beam conditioning optics includes two half-wave plates, a polarizer, two 90° mirrors, a photodetector, an aperture, and a plano-convex lens. The laser is attenuated using the two half-wave plates and the polarizer. The first half wave plate converts the vertical polarization of the laser into horizontal and vertical components. The polarizer removes the vertically polarized light, and the second half wave plate transforms the horizontally polarized light back into vertically polarized light. The laser is then reflected off a 90° mirror and through a ceramic slit to produce a near-uniform “top hat” fluence profile. A plano-convex lens is then placed halfway between the slit and probe volume to achieve a 1:1 magnification. Finally, a second 90° mirror is used to direct the laser beam into the sample cell, with the final cross-sectional area at the probe volume approximately 2 mm × 1.2 mm. Additionally, a third movable 90° mirror is placed between the lens and aperture that redirects the laser beam into a power meter (Coherent FieldMax II TOP) to measure the laser fluence.

The sample cell windows are made of fused silica glass and installed at Brewster’s angle to minimize reflective losses. The laser beam inlet and outlet are aligned along a straight path, while the collection optics are aligned at 35° relative to the laser beam direction. The aerosol inlet and outlet are oriented perpendicular to the laser beam path, with the inlet located at the top and the outlet at the bottom of the cell. The probe volume is defined by the intersection of the laser beam and the detection optical path as defined by the collection optics, consisting of two 50 mm diameter achromatic lenses with focal lengths of 210 mm and 100 mm. The radiation emitted by particles within the probe volume is then focused on a 40 mm diameter lens and imaged onto a fibre optic cable to be transmitted to the detection apparatus.

Figure 2b shows the schematic of the detection apparatus. The apparatus consists of four photomultiplier tube (PMT) assemblies, three dichroic mirrors, and a wheel of neutral density filters, which was not used in this experiment. Incident light is distributed into four paths using three dichroic mirrors with cutoff wavelengths at 490 nm, 567 nm, and 685 nm respectively. The light is then directed to the four PMTs, equipped with bandpass filters centred at 447 nm, 545 nm, 645 nm, and 747 nm, with spectral widths of ± 16 nm, ± 32.5 nm, ± 45 nm, and ± 35 nm, respectively. The PMT voltages are digitized and recorded using a digital oscilloscope (HDO6104 1 GHz High-Definition Oscilloscope−Teledyne) with a 50 W coupling. The oscilloscope is externally triggered using the photodetector as shown in Fig. 2a and records voltages from all four PMTs at a frequency of approximately 500 MHz. The system is calibrated according to the procedure developed by Mansmann et al. [20] and described in the supplemental information (SI).

The aerosol outlet is then connected to the second semi-permeable filter where the rGO is collected and the argon gas is exhausted. In principle, this would allow for *S*_sp_ to be measured in real-time using TiRe-LII, as attempted in this experiment. Additionally, *S*_sp_ is derived offline via Brunauer-Emmett-Teller (BET) specific surface area analysis on the collected rGO powder. Each reduction temperature is analyzed with 10 runs, with each run constituting 400 TiRe-LII traces.

### Incandescent signal temperature analysis

The TiRe-LII signals are first smoothed using a combination of median filtering and locally estimated scatterplot smoothing (LOESS) filtering to minimize the noise in the signal. The original and smoothed traces are shown in the supplemental information. The noise consists of electrical noise, shot noise, and noise from the q-switch of the laser. The signals are then interpreted by fitting the decay curves with a measurement model that connects the particle size distribution with the observed incandescence decay rate. If the particles are modeled as uniformly sized spheres, the modelled intensity is represented by [21]:

1 $${J_{{\mathrm{mod,\boldsymbol{\uplambda}}}}}\left( t \right)={C_{\mathrm{\boldsymbol{\uplambda}}}}{n_{\mathrm{p}}}\frac{{\pi d_{{\mathrm{p}}}^{2}}}{4}{Q_{{\mathrm{abs,\boldsymbol{\uplambda}}}}}\left( {{d_{\mathrm{p}}},{m_{\mathrm{\boldsymbol{\uplambda}}}}} \right){I_{{\mathrm{\boldsymbol{\uplambda},b}}}}\left[ {{T_{\mathrm{p}}}\left( {t,{d_{\mathrm{p}}}} \right)} \right]$$

where *C*_λ_ is a calibration constant, *n*_p_ is the number density of the particles, *d*_p_ is the diameter of the particle, *Q*_abs,λ_ is the spectral absorption efficiency of the particle, *m*_λ_ is the complex refractive index, *I*_λ,b_ is the blackbody spectral intensity, and *T*_p_ is the particle temperature. This formulation assumes a uniform particle size distribution. While this assumption is unlikely to hold in practice, the particle size distribution is unknown and is therefore taken to be uniform for this initial measurement. The calibration constant can be further combined with the particle size and number density to form a general coefficient that is expected to be constant as a function of time.

Equation (1) may then be inverted to recover an instantaneous particle temperature by minimizing the residual between measured and modeled incandescence signals at the four detection channels, provided the wavelength-dependent terms are known. For particles that absorb and emit in the Rayleigh regime, the absorption efficiency is given by

2 $${Q_{{\mathrm{abs,\boldsymbol{\uplambda}}}}}\left( {{d_{\mathrm{p}}},{m_{\mathrm{\boldsymbol{\uplambda}}}}} \right)= - 4x\operatorname{Im} \left( {\frac{{m_{\lambda }^{2} - 1}}{{m_{\lambda }^{2}+2}}} \right)=4xE\left( {{m_\lambda }} \right)$$

where *E*(*m*_λ_) is the absorption function and *x* = π*d*_p_/λ is the size parameter. Particles absorb in the Rayleigh regime when the particles are comparable in size or smaller than the wavelength of light (*x* ≲ 1) and *x*|*m*_λ_| < 1; these conditions ensure that the electronic dipoles that constitute the particle oscillate in phase with an incident electromagnetic wave and therefore absorb and emit independently of each other. This treatment can be extended to larger particles through Rayleigh-Debye-Gans (RDG) theory [22] and has been shown to apply for both soot particles [23] and FLG particles [24]. A key feature of RDG theory is that the absorption cross-section of the particle is insensitive to the specific particle morphology but depends only on the number density of oscillators (amount of matter) contained within the probe volume. Consequently, the pyrometrically-inferred temperature should be insensitive to particle morphology.

**Fig. 3:**
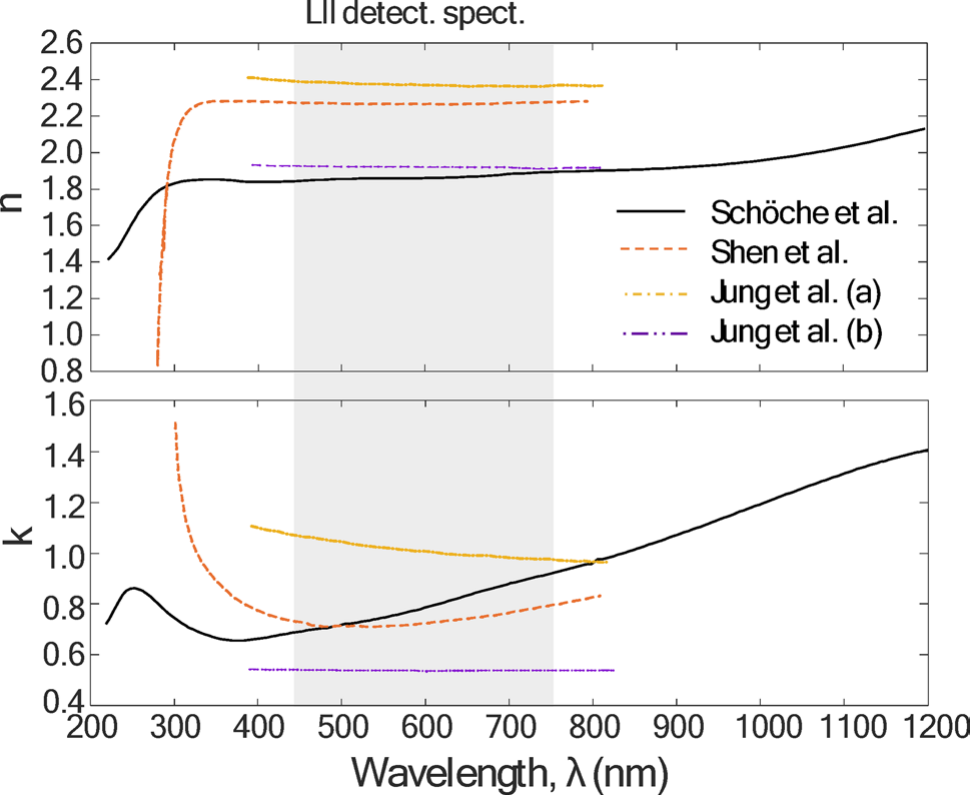
**a** Real and (**b**) imaginary components of the complex refractive index for rGO films and stacks of films determined by ellipsometry, as reported in the literature. These results suggest that the optical properties of rGO are approximately wavelength-independent over the LII detection spectrum [26–28]

**Fig. 4:**
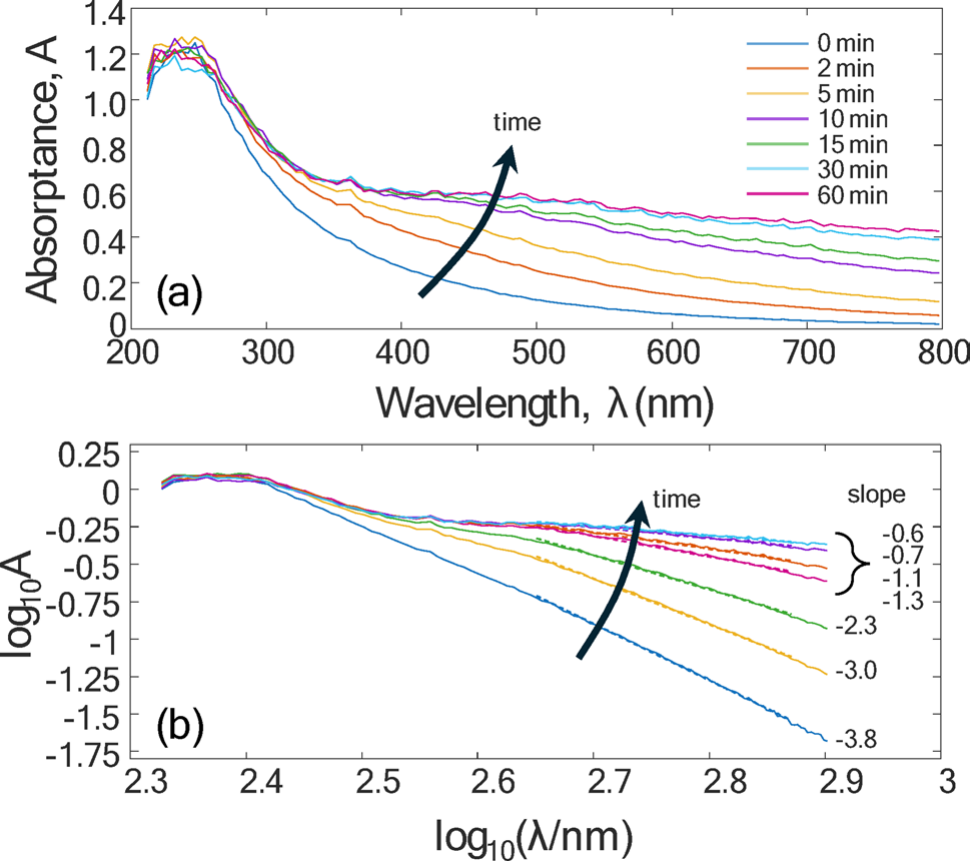
**a** Absorptance of colloidal rGO suspensions measured at various intermediate times during chemical reduction.** b** Absorptance data plotted on a log-log plot to obtain the Ångstrom coefficient over the LII detection spectrum

Several studies have examined the optical properties of rGO films using ellipsometry [25–27] which show a peak at ~ 250 nm corresponding to a π→ π^*^ interband transition and are comparatively flat over the LII detection spectrum (450–750 nm), due to Drude-like behavior (Fig. 3). This observation is further supported by transmittance measurements made on colloidal suspensions of rGO particles at various stages of chemical reduction. The powder was dispersed in 5 mL of Milli-Q (MQ) water to form a 0.5 wt% colloid, and hen ultrasonicated and centrifuged to remove any unexfoliated GO. While the aerosolized GO is thermally reduced using a tube furnace, the colloidal GO was chemically reduced using a solution of 10 mL MQ water, 10 µL of hydrazine solution (35 wt% in MQ water) and 75 µL of ammonia solution (28 wt% in MQ water). The solution was agitated and then placed in a water bath at 100 °C for one hour. At various time intervals, a portion of the colloid was extracted, diluted with MQ water at a 1:1 ratio, and measured using a UV-Vis spectrometer (ThermoFisher Scientific Evolution 300). Figure 4a shows the absorptance, while Fig. 4b shows the results on a log-log plot, where the negative slope of a linear regression line corresponds to the Ångstrom coefficient. For particles that absorb light in the Rayleigh/RDG regime, an Ångstrom coefficient of unity denotes optical properties that are independent of wavelength. Figure 4b shows that the Ångstrom coefficient gradually drops from 3.63 (GO) to a slope near unity by the time the particles are fully reduced.

### Derivation of the specific surface area

The pyrometric temperature decay rate is then used to infer the *S*_sp_. Provided the peak particle temperature does not approach the sublimation temperature of the particle material, the particle cooling rate is dominated by conduction heat transfer [28]

3 $${m_{\mathrm{p}}}{c_{\mathrm{p}}}\frac{{d{T_{\mathrm{p}}}}}{{dt}}= - {q_{{\mathrm{cond}}}}\left[ {{T_{\mathrm{p}}}\left( t \right),{T_{\mathrm{g}}}} \right]$$

When the particle is much smaller than the mean free molecular path in the gas heat conduction takes place in the free molecular regime (FMR), in which gas molecules travel ballistically between the equilibrium gas at *T*_g_ and the particle surface at *T*_p_. Under these conditions the conduction heat transfer rate is given by

4 $${q_{{\mathrm{cond,FMR}}}}={N^{\prime\prime}_{\mathrm{g}}}{A_{{\mathrm{cond}}}}\left\langle {{E_{{\mathrm{g,o}}}} - {E_{{\mathrm{g,i}}}}} \right\rangle $$

where *N*_g_″ is the incident molecular number flux, *A*_cond_ is the particle surface area available for conduction, and < *E*_g, o_–*E*_g, i_> is the average energy change undergone by a gas molecule when it scatters from the particle surface. The latter quantity may be written in terms of the thermal accommodation coefficient (TAC), α, which defines the fraction of the average energy transferred when a gas molecule scatters from the particle surface compared to the amount that would occur if each incident gas molecule reached thermal equilibrium with the particle before it is reemitted into the gas. For a monatomic gas (here argon),

5 $${\left\langle {{E_{{\mathrm{g}},{\mathrm{o}}}} - {E_{{\mathrm{g}},{\mathrm{i}}}}} \right\rangle _{\hbox{max} }}=2{k_{\mathrm{B}}}\left( {{T_{\mathrm{p}}} - {T_{\mathrm{g}}}} \right)$$

where *k*_B_ is Boltzmann’s constant. The incident gas flux is given by *N*_g_″ = *n*_g_*c*_g_/4, where *n*_g_ = *p*_g_/(*k*_B_*T*_g_) is the number density of the equilibrium gas and *c*_g_ = [(8*k*_B_*T*_g_)/(π*m*_g_)]^1/2^ is the mean thermal speed, where *m*_g_ is the molecular mass of the gas. With these substitutions, Eq. (3) becomes

6 $${m_{\mathrm{p}}}{c_{\mathrm{p}}}\frac{{d{T_{\mathrm{p}}}}}{{dt}}= - \alpha {A_{{\mathrm{cond}}}}{p_{\mathrm{g}}}\sqrt {\frac{{2{k_{\mathrm{B}}}}}{{\pi {m_{\mathrm{g}}}{T_{\mathrm{g}}}}}} \left[ {{T_{\mathrm{p}}}\left( t \right) - {T_{\mathrm{g}}}} \right] \cdot t$$

Equation (6) is a separable ordinary differential equation having a solution

7 $$\ln \left[ {\theta \left( t \right)} \right] - \ln \left[ {{\theta _{\hbox{max} }}\left( t \right)} \right]=\frac{{{A_{{\mathrm{cond}}}}}}{{{m_{\mathrm{p}}}}}\frac{{\alpha {p_{\mathrm{g}}}}}{{{c_{\mathrm{p}}}}}\sqrt {\frac{{2{k_{\mathrm{B}}}}}{{\pi {m_{\mathrm{g}}}{T_{\mathrm{g}}}}}} t$$

where θ(*t*) = *T*(*t*) – *T*_g_. Finally, by defining *S*_sp_ = *A*_cond_/*m*_p_, Eq. (7) may rearranged to give

8 $$\ln \left[ {\theta \left( t \right)} \right]={C_1} \cdot {S_{{\mathrm{sp}}}} \cdot t+{C_2}$$

where the coefficient *C*_1_ is

9 $${C_1}=\frac{{\alpha {p_{\mathrm{g}}}}}{{{c_{\mathrm{p}}}}}\sqrt {\frac{{2{k_{\mathrm{B}}}}}{{\pi {m_{\mathrm{g}}}{T_{\mathrm{g}}}}}} $$

and *C*_2_ = ln[θ_max_(*t*)]. Therefore, the *S*_sp_ may be found by plotting ln[θ(*t*)] versus *t* and performing a linear regression. Note that this approach involves solving only linear equations and is therefore amenable to online analysis.

This calculation requires the specific heat, *c*_p_, of the particles, as well as the TAC, *α*, for the particle/gas combination. Due to the limited information available in the literature for rGO, a value of *c*_p_ = 2100 Jkg^− 1^K^− 1^ as presented by Pop et al. [29] for graphene will be assumed, while the TAC is taken to be 0.41 based on TiRe-LII measurements on soot entrained in argon [30]. A sensitivity study will be conducted to determine the potential uncertainty in *S*_sp_ introduced by the imperfectly known parameters.

A further source of uncertainty concerns whether the particles cool in the free molecular regime. This condition is satisfied if the gas molecules travel ballistically between a region in the gas that is in thermal equilibrium at *T*_g_ and the particle surface at *T*_p_. In this sense, the *S*_sp_ is based on a “projected” or “ballistically accessible” particle surface area. The viability of this treatment may be assessed based on the Knudsen number, *Kn* = λ_MFP_ /*L*_eff_, where λ_MFP_ is the mean free molecular path in the gas and *L*_eff_ is a characteristic particle length scale (e.g., the diameter, in the case of a spherical particle). For free molecular conditions to prevail, *Kn* ≳ 1. For argon at normal temperature and pressure, λ_MFP_ ≈100 nm based on its collision diameter, whereas electron micrographs of rGO particles synthesized following a procedure identical to the one in this study reveal sheet widths between 500 nm and 1.5 μm as shown in Fig. 4. Using this length as *L*_eff_ results in a Kn between 0.1 and 0.2, indicating that conduction likely takes place in the transition regime, although the exact characteristic length appropriate for 2D materials is not immediately clear. Accordingly, the FMR approximation likely overestimates the true heat transfer rate since it does not account for collisions between gas molecules in the vicinity of the laser-heated particle that inhibit transfer between the particle surface and the equilibrium gas.

Therefore, a second analysis is done to account for transition-regime effects. While Liu et al. [31] advocate the use of the Fuchs’ boundary sphere method [32], the nonlinearity of this approach is poorly suited to online analysis. Instead, we adopt the McCoy & Cha model [33],

10 $${\dot {q}_{{\mathrm{MCC}}}}=\frac{{{{\dot {q}}_{{\mathrm{FM}}}}}}{{1+\frac{2}{{KnG}}}}$$

where *G* = ${{15 \cdot \alpha } \mathord{\left/ {\vphantom {{15 \cdot \alpha } 2}} \right. \kern-0pt} 2}$ for a monoatomic gas, such as argon. Substituting this result into Eq. (8) yields

11 $${S_{{\mathrm{sp,MCC}}}}= - \frac{{d\left[ {\ln \left( {{T_{\mathrm{p}}} - {T_{\mathrm{g}}}} \right)} \right]}}{{dt}}\frac{{{c_{\mathrm{p}}}4{k_{\mathrm{B}}}{T_{\mathrm{g}}}}}{{{\alpha _{\mathrm{T}}}{k_{\mathrm{B}}}{p_{\mathrm{g}}}\sqrt {{{8{k_{\mathrm{B}}}{T_{\mathrm{g}}}} \mathord{\left/ {\vphantom {{8{k_{\mathrm{B}}}{T_{\mathrm{g}}}} {\left( {\pi {m_{\mathrm{g}}}} \right)}}} \right. \kern-0pt} {\left( {\pi {m_{\mathrm{g}}}} \right)}}} \left( {2+\frac{{{\zeta _{{\mathrm{rot}}}}}}{2}} \right)}}\frac{1}{{1+\frac{2}{{KnG}}}}$$

### Brunauer-Emmet-Teller specific surface area (BET- S_sp_) analysis

Reduced graphene oxide was collected from the semi-permeable membrane at each reduction temperature and characterized ex-situ for *S*_sp_ using a BET surface area analyzer (Micromeritics Gemini VII 2390a) with sample masses of approximately 0.01 g. Prior to analysis, the powder was degassed at 150 °C for 17 h to remove any residual impurities. Following degassing, the sample was loaded into the analyzer, which operates using the static volumetric method as specified in ASTM D6556-21 [34]. In this method, nitrogen gas is flowed into both a sample tube containing the rGO powder and a reference tube at a defined rate. Transducers maintain equal pressure between the tubes while nitrogen adsorbs onto the surface of the sample. The difference in gas volume between the sample and reference tubes is then used to calculate the porosity and *S*_sp_ of the material.

## Results and discussion

### GO powder characterization

**Fig. 5:**
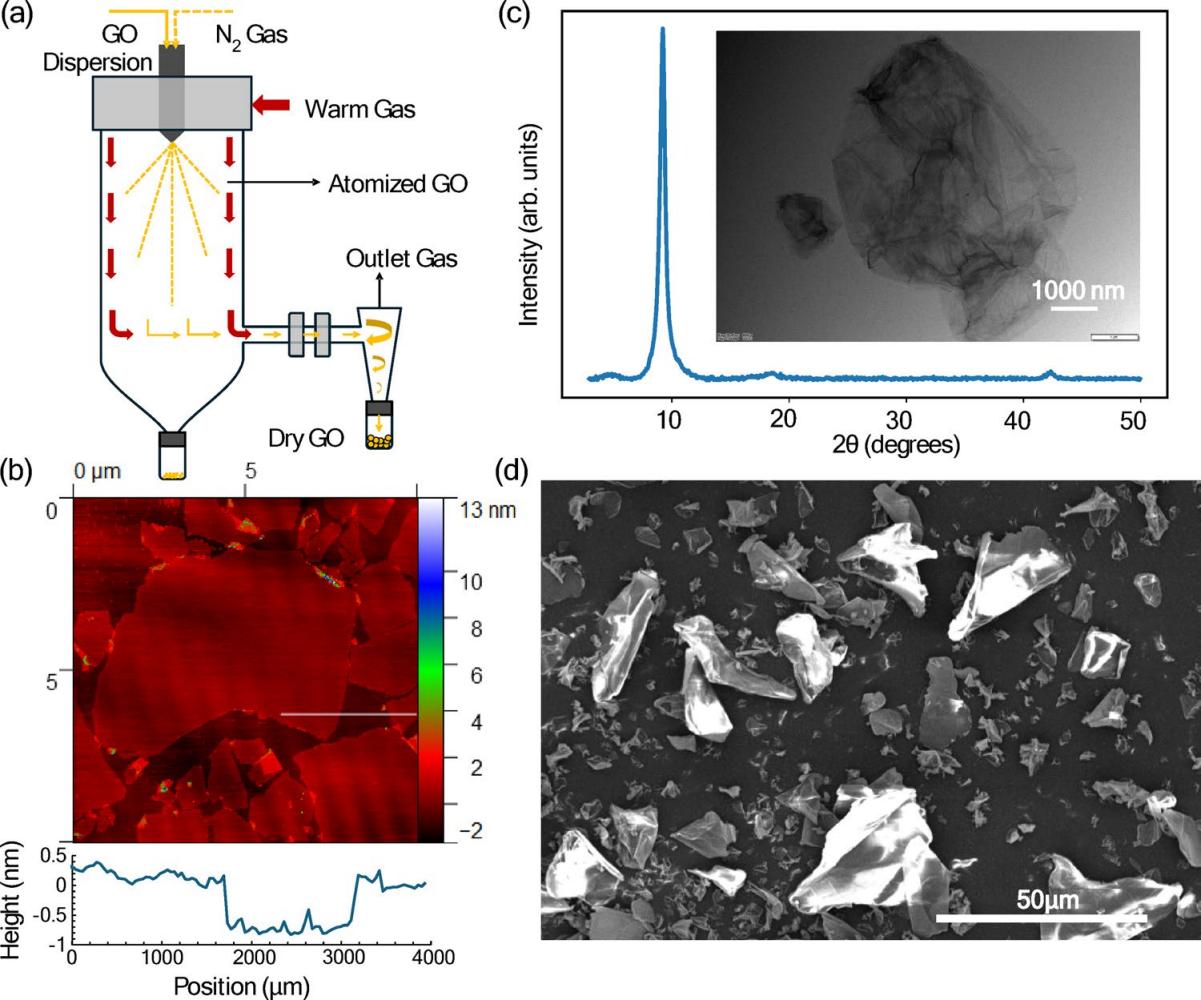
GO precursor dispersion and powder characterization:** a** Schematic of spray drying process to convert 12 g/L GO dispersion in ethanol to powder where a GO dispersion;** b** AFM height image of graphene oxide dispersion prior to spray drying with example height profile (white line) beneath;** c** X-ray diffraction profile of powder produced by process with inset TEM image;** d** SEM image illustrating particle size distribution of powder

To obtain GO powder for exfoliation, the GO slurry in ethanol resulting from the post Hummer’s method washing process was spray dried as shown in Fig. 5a. To confirm the quality of the prepared GO, Fig. 5b shows an AFM image of the GO from this dispersion coated onto mica. The sheets are several micrometers in lateral size and 0.7–1 nm in thickness as is characteristic of exfoliated GO. After spray drying, the resulting powder is comprised of reaggregated or restacked sheets which exhibit crystalline features as observed by powder XRD, shown in Fig. 5c. The reflection at 2q = 9.79° corresponds to the d-spacing of the GO powder which is calculated using Bragg’s law to be 9.02 Å. The average crystallite size, determined using the Scherrer equation, was found to be 92.1 Å. The inset of Fig. 5c shows a TEM image where multiple thin sheets appear to be wrinkled and folded on top of one another. To obtain a better overview of the powder aggregate size and morphology SEM imaging was carried out as shown in Fig. 5d. The irregularly shaped particles range in size from several microns to 30–40 μm. The BET surface area of this powder was found to be 28.8 m^2^/g.

### Effective particle temperature

**Fig. 6:**
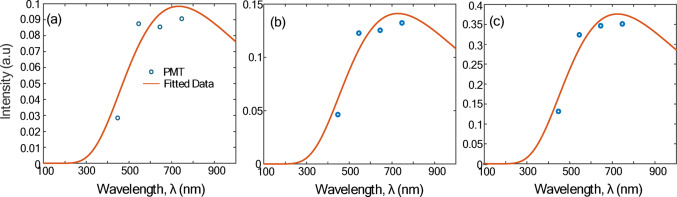
Pyrometric fits at the peak intensity for reduction temperature of:** a** 500 °C,** b** 900 °C, and** c** 1300 °C

**Fig. 7:**
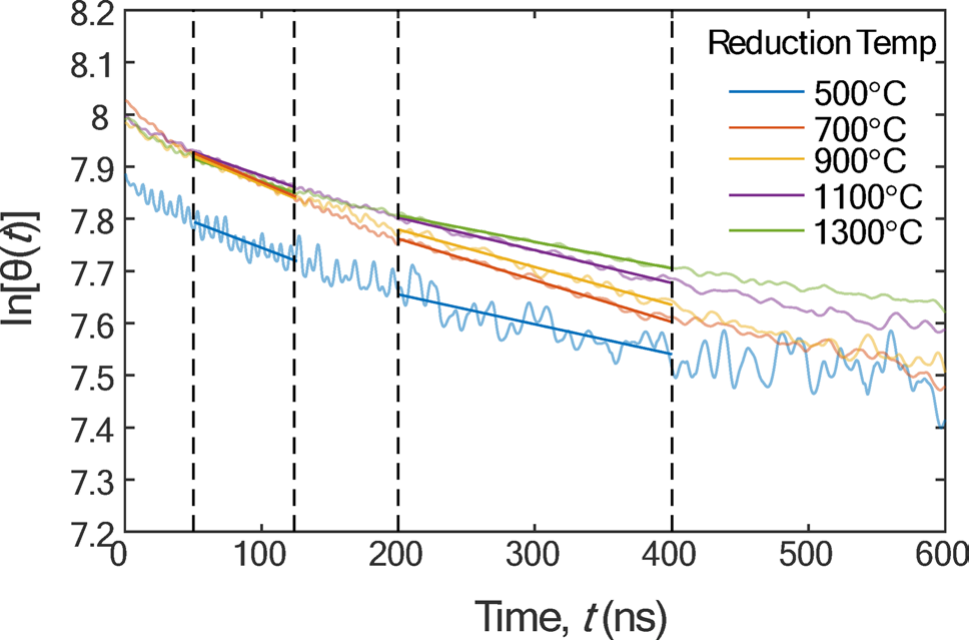
Linear regression of ln[θ(t)] versus time for different reduction temperatures

After each experiment, four sets of intensity signals over time were collected at wavelengths of 447 nm, 550 nm, 650 nm, and 747 nm, respectively. The signals were averaged over 400 shots and then conditioned as described in Sect. 2.3, and the pyrometric temperature was calculated at each instant by fitting Eq. (1) to the four intensity measurements. Figure 6 shows sample fits for particles reduced at set-points of 500 °C, 900 °C, and 1300 °C, while Fig. 7 shows the temperature decay curves plotted on a semilog plot. The original and filtered temperature decay curves are in Figures B-2 and B-3 of the supplemental information.

### Specific surface area

**Fig. 8:**
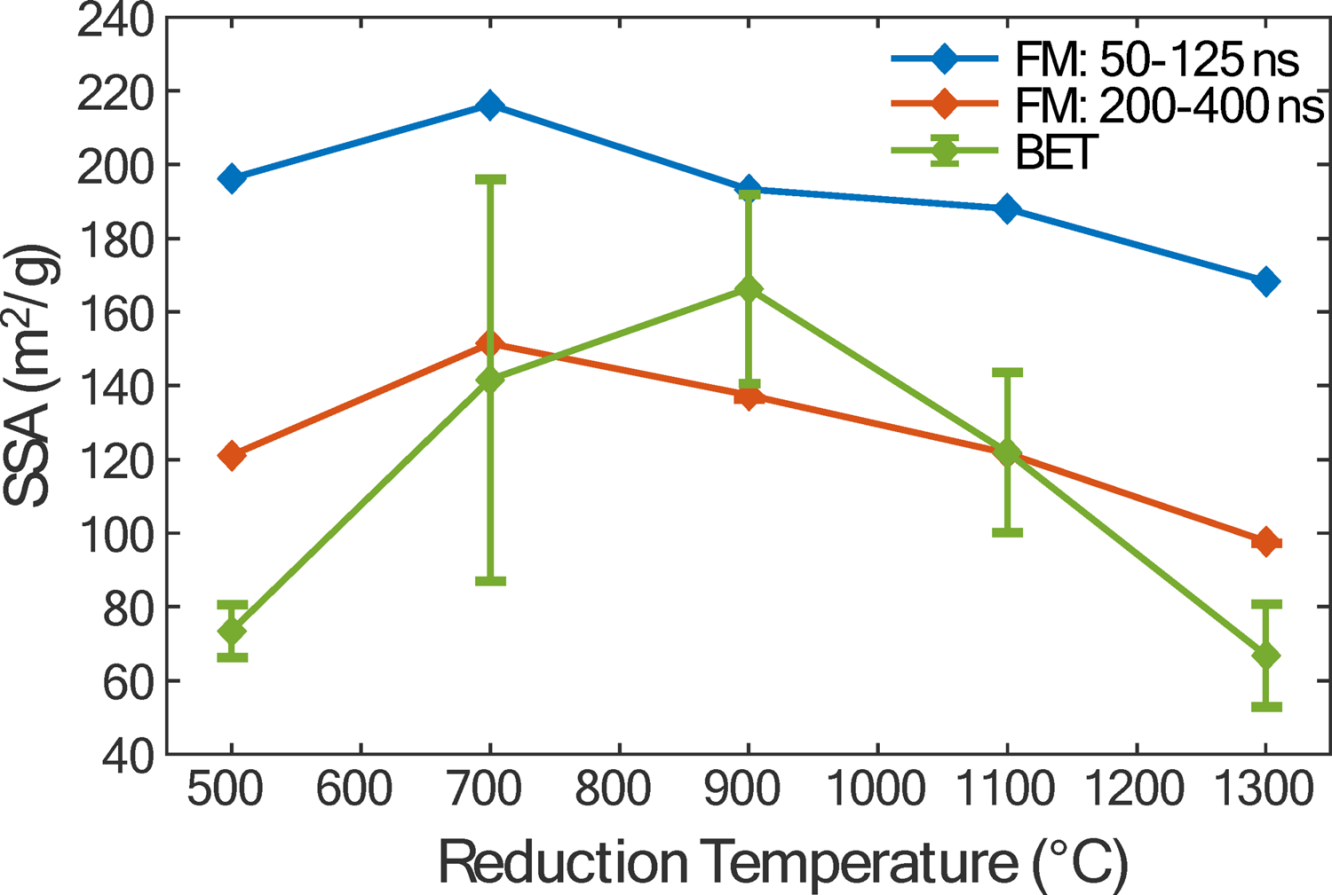
Specific surface area versus reduction temperature derived from time-resolved laser-induced incandescence assuming conduction cooling described by the free-molecular (FM) regime for ranges 50–125 ns and 200–400 ns and from ex-situ BET measurements. The vertical error bars for the BET correspond to 1 mg per mass of rGO measured

Figure 7 shows a plot of ln[*θ*(*t*)] versus time, which is expected to be linear due to the exponential decay characteristics of conduction cooling. In principle, this slope, dln[*θ*(*t*)]/d*t*, is used to calculate *S*_sp_ via Eq. (8) and is constant after an initial abrupt change in cooling rate (~ 10–20 ns), which is similar to the commonly-observed and heretofore unexplained anomalous cooling phenomenon in TiRe-LII experiments on soot and other nanoparticles [30]. Additionally, the temperature decay becomes progressively nonexponential due to nonuniform cooling of different sized particles [35]. Therefore, an appropriate interval must be chosen over which the decay is exponential. In contrast to TiRe-LII measurements on soot and other materials, in which the slope is unambiguous (e.g., [30]), Figure 7 shows that the slope changes around 125 ns, transitioning from one exponential decay slope to another. This change is unexpected and is not attributed to anomalous cooling or polydispersity effects, as it occurs after 20 ns and before polydispersity effects are expected to manifest. A possible explanation is that the discontinuous slope results from a laser-induced change in particle morphology or optical properties, for example due to further particle reduction. To account for this uncertainty, Fig. 8 shows the *S*_sp_ as a function of reduction temperature, derived using the free molecular (FM) model based on d*θ*/d*t* values from the time ranges of 50–125 ns and 200–400 ns compared with results from *ex-situ* BET sampling. For the BET measurements, a conservative error estimate of 1 mg is applied for each sample. The slope calculated over the 50–125 ns range yields *S*_sp_ values exceeding 200 m^2^/g, whereas the 200–400 ns range yields lower values, only slightly above 100 m²/g. Error bars for the LII-derived *S*_sp_ parameters are excluded for clarity, but they are shown in Table 1 for the 200–400 ns interval, assuming 10% uncertainty in *c*_p_ and α. These uncertainties are much larger than the uncertainties introduced by noise sources and dominate the overall uncertainty in the LII-derived quantities.

**Fig. 9:**
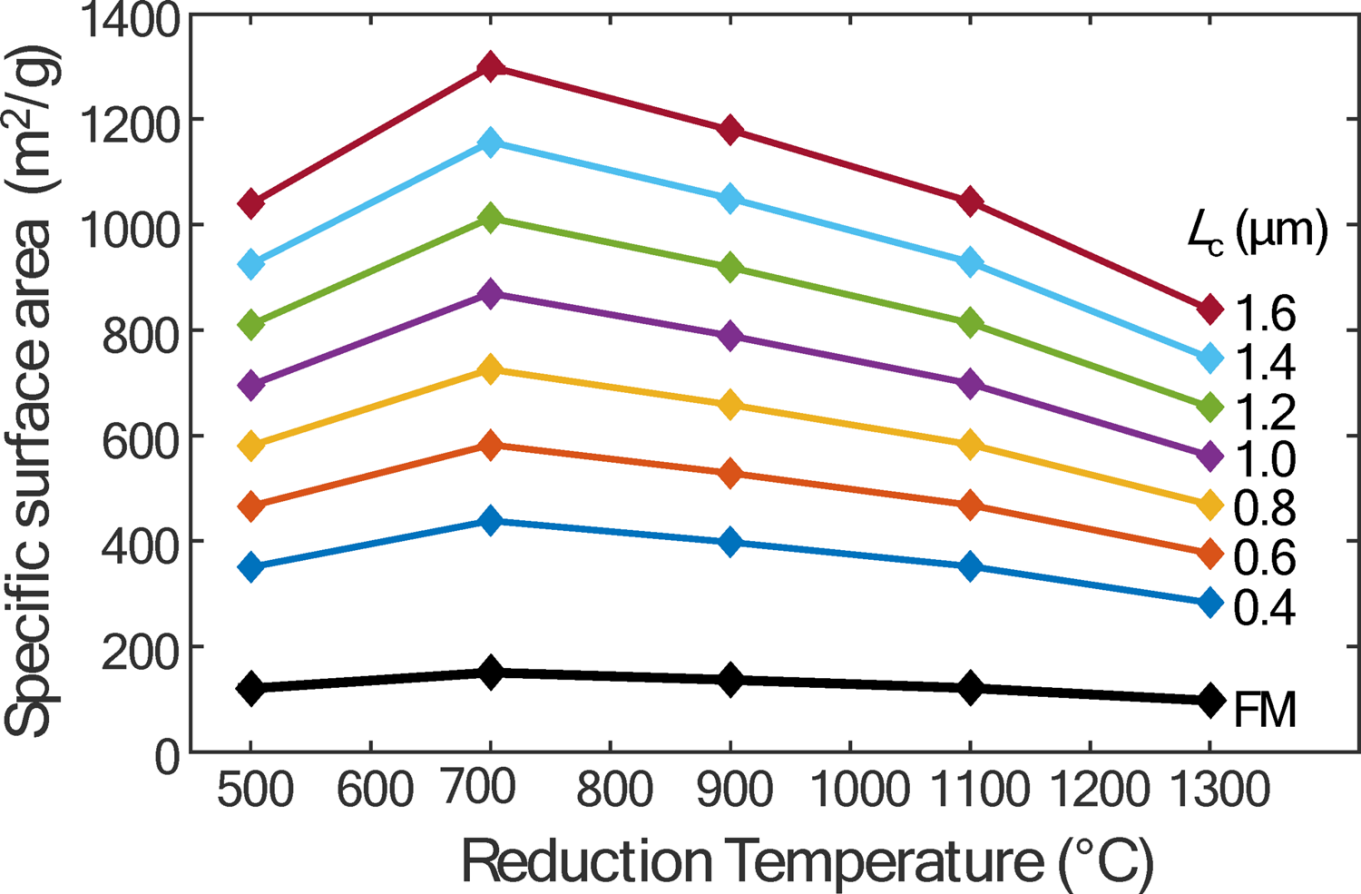
TiRe-LII derived S_*sp*_ versus reduction temperature derived from assuming transition regime conduction cooling between 200–400 ns for different assumed characteristic lengths

While there is some ambiguity as to which portion of the cooling curve should be used to infer the *S*_sp_, Fig. 9 shows that both intervals provide the same trends in *S*_sp_ with respect to reduction temperature. These curves show that *S*_sp_ peaks at an intermediate reduction temperature, which is consistent with previous work by Huh [36] and Sengupta et al. [14]. Based on Fourier-transform infrared (FTIR) analysis, these studies suggested the initial increase in *S*_sp_ results from the removal of hydroxyl (-OH) and carboxyl (-COOH) groups. Since the removal of these groups does not require carbon atoms from the graphitic lattice, no structural defects are introduced. However, at temperatures beyond a critical threshold, epoxy (COC) and carbonyl (C = O) groups are removed from the rGO particles, leading to in-plane C = C bond breakage and defect formation, which reduces *S*_sp_. Huh [36] supports this hypothesis with TEM images, showing that at even higher reduction temperatures, the originally crumpled rGO particles begin to unfold and restack, forming more graphite-like structures and reducing the overall surface area.

**Table 1:** Sensitivity of the inferred S_sp_ to uncertainty in specific heat and thermal accommodation coefficient using the free molecular regime over the 200–400 Ns time range

Reduction temp (°C)	Nominal (m^2^/g)	± 10% c_*p*_ (m^2^/g)	∓ 10% α (m^2^/g)	Combined (m^2^/g)
500	121	[109, 133]	[110, 135]	[103, 139]
700	151	[136, 167]	[138, 168]	[129, 174]
900	137	[123, 151]	[125, 153]	[117, 158]
1100	122	[109, 134]	[111, 135]	[103, 139]
1300	98	[88, 108]	[89, 109]	[83, 112]

It is worth noting that while both Sengupta et al. [14] and Huh [36] report similar trends in *S*_sp_ with reduction temperature, they report different critical temperatures. Sengupta et al. [14] report significant changes in the FTIR spectra at reduction temperatures between 300 °C and 400 °C, implying a critical temperature of ~ 350 °C, while Huh [36] observed a broader transition range and a critical temperature near 800 °C. These differences could be due to differences in both the GO precursor and the reduction methods between these studies. Sengupta et al. [14] used GO synthesized via a modified Tour method, while Huh [36] employed the modified Hummers’ method. Different oxidation processes are known to produce GO with significantly different oxygen content and functional group compositions [18, 37, 38].

These studies also used different approaches to heat the aerosol. Sengupta et al. [14] preheated the furnace to the target reduction temperature and maintained it for 30 min, whereas Huh [36] gradually increased the temperature from room temperature at a rate of 5 °C/min. These methodological differences likely influenced the reduction temperature ranges. Nevertheless, the consistent trend observed across both studies suggests that this behavior is common to various types of rGO and reduction methods, including the approach used in this work.

Zhao et al. [39] produced rGO by heating GO sheets within a horizontal tube furnace at 5 °C/min and reduction temperatures between 200 and 900 °C. They observed that the *S*_sp_ increased from 12 m²/g to a peak of 227.05 m²/g at 500 °C, before dropping to 154 m²/g at 900 °C. Using the *S*_sp_ found between 200 and 400 ns, the approximate difference in morphology and composition can be identified by comparing with Huh’s [36] results. In this study, the *S*_sp_ increases from 121.1 m²/g to 151.5 m²/g between 500 °C and 700 °C, which can be attributed to the defect-free removal of hydroxyl and carboxyl groups. The critical temperature of 700 °C observed here is consistent with the value reported by Huh [36]. Beyond this temperature, *S*_sp_ begins to decrease. The initial drop between 700 °C and 900 °C, from 151.5 m²/g to 137.4 m²/g, is likely due to the removal of carbonyl and epoxy groups, which introduces defects into the lattice. Between 900 °C and 1300 °C, although thermal annealing may help repair some defects, the further decrease to 97.7 m²/g is attributed to the unfolding and restacking of rGO sheets, which diminishes the total surface area [39]. Figure 8 also shows that the BET results closely align with the FM results in terms of magnitude, although the BET results suggest a critical temperature of 900 °C in the BET results, compared to 700 °C from the LII-derived *S*_sp_.

While the BET and LII results both suggest a similar *S*_sp_ magnitude (~ 100 m^2^/g), this is considered to be atypically low for rGO particles. Previous studies have reported *S*_sp_ values for rGO in the range of 100–600 m²/g [40, 41]. Additionally, Dudding et al. [42] performed batch exfoliation using the same GO precursor and found *S*_sp_ values between 350 and 550 m^2^/g at various temperatures. This result suggests that the thermal reduction process used in this study may have been inefficient, potentially due to combustion or degradation from leaks in the system. Nonetheless, the strong agreement between the trends and relative magnitudes of the BET and LII results in this study demonstrates the potential of TiRe-LII as a diagnostic tool for real-time measurement of rGO *S*_sp_.

### Potential transition-regime effects

**Fig. 10:**
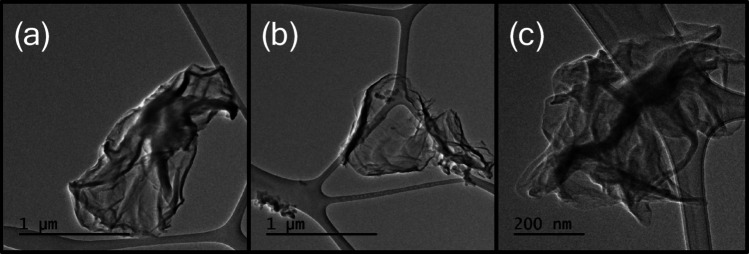
TEM images of drop cast rGO samples:** a** 1.5 μm** b** 1 μm, and** c** 500 nm collected at the exit of the continuous flow reactor operated at identical conditions as described in this study

Although the assumption of free molecular heat conduction regime produces specific surface areas that are aligned with those found with BET analysis, one would expect heat conduction to occur within the transition regime based on the micrographs shown in Fig. 10.

Since the appropriate characteristic length for the 2D particles in this study are unknown, the *S*_sp_ is recalculated using a range of characteristic lengths (0.4–1.6 μm). Figure 9 shows the *S*_sp_ obtained from the TiRe-LII data over the 200–400 ns interval analyzed using a transition regime model compared to values found assuming free molecular conductive cooling. While the overall trends are maintained, the *S*_sp_ values increase with the characteristic length as *Kn* drops. Consequently, as intermolecular collisions increasingly impede gas molecule motion between the particle and the surrounding equilibrium gas, a higher *S*_sp_ is required to achieve the observed pyrometric temperature decay rate. However, the *S*_sp_ values predicted by the MCC model at all of the candidate characteristic lengths are higher than those reported in the literature and are not consistent with expectations for rGO. This discrepancy cannot be explained solely by variations in density and specific heat, which suggests that the relevant characteristic length that governs diffusion transport between the particle and the surrounding gas is not simply the lateral length of the rGO particle visible in the micrographs. This finding highlights the need for further research using tools like direct simulation Monte Carlo (DSMC) to examine sphericity, flatness and elongation to better determine an appropriate characteristic length [31].

## Conclusions

Reduced graphene oxide (rGO) is a promising alternative to graphene that is amenable to high yield production. One of the most effective methods of rapid synthesis is tube furnace thermal reduction. However, the properties of the synthesized rGO particles depend heavily on the morphology of the graphene oxide (GO) particles and the reduction parameters. Consequently, real-time characterization of the functional properties of rGO particles is crucial for industrial-scale production.

This study investigates the use of time-resolved laser-induced incandescence (TiRe-LII) to characterize the specific surface area (*S*_sp_) of rGO in real time. A TiRe-LII apparatus was integrated online with a tube furnace thermal reduction system to allow for real-time measurements of the synthesized rGO particles. Incandescent signals were recorded and processed between two different time ranges, 50–125 ns and 200–400 ns, to provide *S*_sp_ values. The data was analyzed initially assuming conduction between the laser-heated particles and the surrounding gas took place in the free molecular (FM) regime, and subsequently accounting for potential transition regime effects using the McCoy-Cha (MCC) model. Ex-situ characterization was also carried out on the rGO powder via a Brunauer–Emmett–Teller (BET) analysis.

The *S*_sp_ values derived from TiRe-LII using the FM regime were consistent in both magnitude and trend with those found using BET- *S*_sp_ analysis. When accounting for transition-regime effects, the LII-inferred *S*_sp_ values were nearly an order-of-magnitude higher than the BET-values, but more consistent with *S*_sp_ values reported from other processes in the literature. This discrepancy may be due to uncertainty in the characteristic length used to predict the transition-regime effects, which has yet to be derived for 2D materials like GO and rGO.

Nonetheless, the strong agreement in overall trends between the FM model, MCC model, and BET results indicates that TiRe-LII can provide reliable real-time *S*_sp_ measurements for rGO. Future research should focus on determining the specific heat capacity, density and thermal accommodation coefficient of rGO to improve the robustness of the *S*_sp_ estimates. A major source of uncertainty originates from the ambiguity regarding the region over which the cooling rate decays exponentially, or the linear region when the excess temperature is plotted on a semilog plot (Fig. 7). The presence of two linear regimes having different slopes may be due to changes in the bulk optical properties or particle morphology introduced by the laser pulse, but this requires further investigation, potentially by combined TiRe-LII and line-of-sight attenuation measurements (e.g., [43]).

Additionally, optical measurements, such as *ex-situ* mass absorption cross-section measurements (CERMS) or spectral absorption coefficient measurements using an aethalometer, can elucidate how the morphology and optical properties change as a function of reduction temperature to further refine the TiRe-LII analysis. Further validation of the proposed hypothesis regarding *S*_sp_ changes could be achieved through Fourier-transform infrared (FTIR) spectroscopy and elemental analysis to study the evolution of oxygen-containing functional groups. Transmission electron microscopy and atomic force microscopy may also be performed to provide information on the evolution of morphology and particle sphericity with reduction temperature.

## Supplementary Information

Below is the link to the electronic supplementary material.

Supplementary Material 1

## Data Availability

The data supporting the findings of this study are available in the Borealis repository at https://borealisdata.ca, with the identifier DOI: https://doi.org/10.5683/SP3/XV8VFW.
