# Supplementary material for: In situ measurement of the specific surface area of reduced graphene oxide using time-resolved laser induced incandescence
Source: Appl Phys B. 2026 Apr 11;132(5):60. doi: 10.1007/s00340-026-08633-0 (PMC13068760; doi:10.1007/s00340-026-08633-0)
Supplement: Supplementary file 1 — Supplementary Material 1 [file 340_2026_8633_MOESM1_ESM.docx]

**In situ measurement of the specific surface area of reduced graphene oxide using time-resolved laser induced incandescence**

***Supplemental Material***

Horace I. Looi^1^, Sarah Jahnkani^2^, Michael Pope^2^, and Kyle J. Daun^1,^^[[1]](#footnote-1)^*

^1^*Department of Mechanical and Mechatronics Engineering, University of Waterloo, Waterloo, ON, Canada*

^2^*Department of Chemical Engineering, University of Waterloo, Waterloo, ON, Canada*

**Appendix A:** **TiRe-LII relative calibration procedure**

The TiRe-LII system was calibrated for relative intensity as opposed to absolute intensity using the apparatus shown in Figure A-1, following Mansmann et al. [1]. Each photomultiplier tube (PMT) was calibrated for sensitivity using a halogen-tungsten calibration lamp and for gain across multiple gain levels. Light from a calibration lamp (SLS201L/M, Thorlabs) was imaged into a fiberoptic cable, which led to an integrating sphere (∅2″ IS200-4, Thorlabs) designed to create a uniform light source. The integrating sphere was positioned at the LII probe volume, eliminating the need for further realignment. As the PMTs were connected to an oscilloscope with a 50 Ω coupling, precautions were taken according to Table A-1 to ensure that the PMT output voltages did not exceed the recommended maximum output voltages.

Table A-1 PMT maximum output voltage ratings

| **Illumination**  **Time** | **Max Output Voltage**  **(50 Ω coupling)** | **Max Repetition**  **Frequency** |
| --- | --- | --- |
| DC | 5 mV | - |
| 100 µs | 50 mV | 100 Hz |
| 1 µs | 500 mV | 10 Hz |
| 10 ns | 5 V | 1 Hz |

To prevent damage to the PMT, the illumination time is reduced using an optical chopper placed between the integrating sphere and collection optics, increasing the maximum repetition frequency and subsequently allowing a higher voltage readout. The modulated signal also facilitates subtraction of the background signal.

Figure A-1: Apparatus for calibrating the LII detection system.

The calibration factors were determined by establishing a relationship between the induced voltage on a PMT and a known amount of incident irradiation, as well as to examine how this relationship varies across different PMT gains. The irradiance, *E_i_*^ref^, incident on the *i*th PMT within a wavelength bandwidth Δλ is [1]

$$E_{i}^{\text{ref}}=\int_{\Delta\lambda_{i}} I\left(\lambda\right) d\lambda$$

where *I*(λ) is the intensity of the calibration light source. The intensity calibration factor, *D_i_*, is then found from the corresponding PMT voltage,

$$D_{i}=\frac{\tau_{i}E_{i}^{\text{ref}}}{V_{i}^{\text{meas}}}$$

where τ*_i_* is the transmission for the *i*th PMT of the neutral density (ND) filter used. Subsequently, *D_i_* can be normalized against a single PMT to derive a relative intensity calibration factor, *D_i_*^rel^.

The voltage applied on the voltage divider circuit of a PMT is controlled by gain controllers. This allows the PMT to measure a wide range of light levels, thereby increasing its dynamic range. However, although the general relationship between voltage gain and the induced PMT signal is well defined, a correction must be applied to derive the exact relationship due to variations in components. This correction can be achieved by determining a gain correction factor, *G_i_*,

$$G_{i}\left(V_{i}^{\text{gain}}\right)=\left(\frac{V_{i}^{\text{gain}}}{V_{i}^{\text{gain,ref}}}\right)^{A_{i}}$$

where *V_i_*^gain^ is the gain voltage for an *i*th PMT, *V_i_*^gain,ref^ is the reference gain voltage at an *i*th PMT, and *A_g,i_* is a coefficient that can be found through a weighted minimization over 11 gain voltages as denoted by *V_i,j_*^gain^ where *j* represents each gain voltage measurement:

$$A_{\text{g},i}=\mathop{\text{argmin}}\limits_{A_{\text{g},i}} \left[\left\|\sum_{j=1}^{11} \frac{V_{i,j}^{\text{meas}}-V_{i}^{\text{meas,ref}}\left(\frac{V_{i,j}^{\text{gain}}}{V_{i}^{\text{gain,ref}}}\right)^{A_{\text{g},i}}}{\sigma_{i,j}^{\text{gain}}}\right\|_{2}^{2}\right]$$

where *V_i,j_*^meas^ represents the *j*th measured voltage for an *i*th PMT, *V_i_*^meas,ref^ represents the reference measured voltage for an *i*th PMT, and *σ_i,j_*^gain^ represents one standard deviation of the *j*th measured voltage for an *i*th PMT.

Using the gain correction factor and relative intensity calibration factor, at the *i*th PMT with an applied voltage, *V_i_*^gain^, the relative intensity is found from the raw measurement according to

$$J_{\text{corr},i}=D_{i}G_{i}\left(V_{i}^{\text{gain}}\right)J_{\text{exp},i}$$

To simplify the description in the remainder of the section, the four PMTs will now be denoted according to Table A-2.

Table A-2: PMT naming convention.

| PMT Name | PMT 1 | PMT 2 | PMT 3 | PMT 4 |
| --- | --- | --- | --- | --- |
| Spectral bandwidth | 431 to 463 nm | 512 to 577 nm | 600 to 690 nm | 722 to 782 nm |

Due to the lower intensity of the halogen-tungsten lamp, calibration could not be performed across the whole gain range (0 to 1000 gain). Instead, it was conducted from 650 to 900 in increments of 25 gain for PMT 1 and PMT 2, while for PMT 3 and PMT4, calibration was performed from 500 to 750 in increments of 25. The change in measured voltage as a function of gain for PMT1 is shown in Figure A-3. The derived *A_i_* and *$D_{i}^{\text{rel}}$*, normalized by PMT1, for each PMT is shown in Table A-3.

Figure A-2: Measured voltage vs gain for PMT 1 – Halogen-Tungsten lamp

Table A-3: Calibration constants derived at each PMT – Halogen-Tungsten lamp

| PMTS | PMT1 | PMT2 | PMT3 | PMT4 |
| --- | --- | --- | --- | --- |
| *A_i_* | 8.5492 | 6.8132 | 6.9645 | 7.2768 |
| *$D_{i}^{\text{rel}}$* | 1 | 6.2665 | 7.2036 | 5.1968 |

The calibration constant *$D_{i}^{\text{rel}}$* and *G_i_*, determined based on the gain voltages used in the study, can be used to modify the intensity signals before fitting to find the pyrometric temperature.

**Appendix B: Smoothing/denoising of pyrometric temperatures**

The incandescence data is generated by averaging over 400 shots. These curves are contaminated with both q-switch noise, visible as a “ringing feature” near the peak signal, as well as photonic shot noise, which becomes more prominent at later cooling times. Noise affected all channels but was particularly prominent on the 645 nm trace. The q-switch noise may be removed partially by subtracting signals made without an aerosol present in the probe volume, but some ringing artifacts remain. Accordingly, the signals were then conditioned using additional median filtering and locally estimated scatterplot (LOESS) smoothing in the MATLAB^®^ environment. Figure B.1 shows the original and conditioned incandescence traces obtained from the 900°C reduction temperature, which are representative of the other traces. Figures B2 and B3 show the pyrometric temperature decay curves obtained using the unprocessed and processed incandescence signals.

Figure B.1: Raw (calibrated) and processed absolute intensity curves for the 900 °C reduction temperature. (a) 447 nm; (b) 545 nm; (c) 645 nm; (d) 747 nm.

Figure B.2 shows the original pyrometric temperature versus time with no modifications to the incandescent signals and Figure B.3 shows the smoothened pyrometric temperature versus time. The signals were smoothened with a combination of q-switch noise removal, median filtering and locally estimated scatterplot smoothing (LOESS) filtering.

Figure B.2: Original pyrometric temperature versus time for five reduction temperatures

Figure B.3: Smoothed pyrometric temperature versus time for five reduction temperatures

**References**

1. R. Mansmann, T. A. Sipkens, J. Menser, K. J. Daun, T. Dreier, and C. Schulz, Appl Phys B 125, (2019).

1. * Corresponding author, kjdaun@uwaterloo.ca [↑](#footnote-ref-1)
